# PGC-1α is Dispensable for Exercise-Induced Mitochondrial Biogenesis in Skeletal Muscle

**DOI:** 10.1371/journal.pone.0041817

**Published:** 2012-07-24

**Authors:** Glenn C. Rowe, Riyad El-Khoury, Ian S. Patten, Pierre Rustin, Zolt Arany

**Affiliations:** 1 Cardiovascular Institute, Beth Israel Deaconess Medical Center, Harvard Medical School, Boston, Massachusetts, United States of America; 2 Hôpital Robert Debré, and Université Paris, Faculté de Médecine Denis Diderot, Paris, France; Mayo Clinic, United States of America

## Abstract

Exercise confers numerous health benefits, many of which are thought to stem from exercise-induced mitochondrial biogenesis (EIMB) in skeletal muscle. The transcriptional coactivator PGC-1α, a potent regulator of metabolism in numerous tissues, is widely believed to be required for EIMB. We show here that this is not the case. Mice engineered to lack PGC-1α specifically in skeletal muscle (Myo-PGC-1αKO mice) retained intact EIMB. The exercise capacity of these mice was comparable to littermate controls. Induction of metabolic genes after 2 weeks of in-cage voluntary wheel running was intact. Electron microscopy revealed no gross abnormalities in mitochondria, and the mitochondrial biogenic response to endurance exercise was as robust in Myo-PGC-1αKO mice as in wildtype mice. The induction of enzymatic activity of the electron transport chain by exercise was likewise unperturbed in Myo-PGC-1αKO mice. These data demonstrate that PGC-1α is dispensable for exercise-induced mitochondrial biogenesis in skeletal muscle, in sharp contrast to the prevalent assumption in the field.

## Introduction

Endurance exercise is a powerful inducer of mitochondrial biogenesis in skeletal muscle [1], [2], [3]. This process is thought to underlie numerous of the benefits seen with endurance exercise. Increases in mitochondrial function have been associated with a reduction in diabetes and obesity outcomes, delayed effects of aging, and improved exercise capacity [3], [4], [5], [6]. Many of these benefits are likely due to increased expression of electron transport chain (ETC) enzymes and oxidative phosphorylation (OXPHOS).

Mitochondrial biogenesis occurs via the coordinated expression of 100s of genes on both the nuclear and mitochondrial genomes [7], [8]. The mitochondrial genome encodes 13 essential subunits of ETC complexes I, III, IV and V, while the nuclear genome encodes the remaining subunits and all subunits of complex II. Members of the nuclear respiratory factor (NRF) and estrogen-related receptor (ERR) families of transcription factors regulate the expression of the nuclear encoded genes [7], [8], [9], while Transcription factor A, mitochondrial (Tfam) regulates the expression of mitochondrial encoded genes [10].

Peroxisome proliferator-activated receptor gamma coactivator-1α (PGC-1α) interacts with a variety of transcription factors to activate broad genetic programs in various tissues. In skeletal muscle, as in other tissues, PGC-1α potently induces mitochondrial biogenesis and oxidative capacity [11], [12], [13], [14], [15], [16]. To do so, PGC-1α coactivates ERRs and NRFs, and induces the expression of Tfam, thus coordinating the expression of genes from both mitochondrial and nuclear genomes. Animals transgenically expressing PGC-1α in skeletal muscle contain highly oxidative myofibers, rich in mitochondria and supporting capillary network [11], [13], [17]. The animals are able to run longer and further in endurance-based training [18], [19]. These animals are also protected from denervation atrophy, and muscle dystrophy [20], [21]. Many of these changes closely mirror the changes seen with endurance exercise. Whole body deletion and muscle-specific deletion of PGC-1α result in mild decrease in exercise performance [22], [23], [24]. PGC-1β, a homolog of PGC-1α, exhibits many of these same properties [18], [25], [26], [27], [28].

PGC-1α mRNA and protein are highly responsive to a variety of environmental signals and intracellular signaling cascades, including cAMP, AMPK, Sirt1, and others [29], [30], [31], [32], [33]. PGC-1α is thus generally felt to be a critical node for signaling to mitochondrial biology [34], [35]. Exercise potently induces the expression of PGC-1α mRNA in skeletal muscle in both humans and rodents [36], [37], [38], [39], [40], [41]. This occurs in large part via the robust activation of an otherwise dormant alternative promoter [35], [42], [43], in part via β2 adrenergic stimulation. In contrast, the expression of PGC-1β is not induced by exercise. These observations, in conjunction with the observation that PGC-1α over-expression in skeletal muscle recapitulates many of the hallmarks of exercise-induced changes, has led to the widespread assumption by us and others that PGC-1α is required for exercise-induced mitochondrial biogenesis (EIMB) [3], [17], [36], [44], [45], [46], [47]. We show here, however, that this is not the case. Using mice genetically engineered to lack PGC-1α in skeletal myocytes, we show that muscle PGC-1α is entirely dispensable for EIMB.

## Materials and Methods

### Ethics Statement

All animal experiments were performed according to procedures approved by the Beth Israel Deaconess Medical Center Institutional Animal Care and Use Committee (Protocol Number 058–2008).

### Animal Experiments

10 to 12-week old female PGC-1α muscle-specific knockout mice (Myo-PGC-1αKO) as previously described [35] and female control littermates were used for all experiments.

### Voluntary Wheel Runs

Animals were subjected to either overnight or 2-week in-cage voluntary running wheel endurance exercise. Exercise performance was measured using electronic monitoring system (VitalView). All mice were housed individually. All study groups received identical chow, were sacrificed and the same time of day, and were controlled for gender.

### Real-Time PCR

Total RNA was isolated from mouse tissue using TRIzol (Invitrogen) method respectively. Samples were reverse transcribed (Applied Biosystems) and quantitative real-time PCR performed on the cDNAs in the presence of fluorescent dye (SYBR green, BioRad). Expression levels were determined using the comparative cycle threshold (2^−ΔΔCt^) method [48].

### Transmission Electron Micrographs

Muscles were dissected, trimmed (1 mm × 2 mm) and placed into fixative. Samples were processed for transmission electron micrographs (TEM) in the BIDMC Electron Micrograph Core using standard procedures. Quantification of EMs was performed computationally, using NIH Image software. Random fields were chosen and the mitochondria area were measured per total area. All quantifications were performed blindly.

### Electron Complex Activity

Muscles were dissected out, and trimmed (1 mm X 2 mm) fragments and snap frozen on liquid nitrogen. Electron transport activity was determined as previously described [49], [50].

### Statistical Analysis

The data are presented as means ± standard error of the mean (SEM). Statistical analysis was performed with Student’s t test for all *in vitro* experiments and ANOVAs for all *in vivo* experiments. *P* values of less than 0.05 were considered statistically significant.

Additional method details on western blotting can be found in Methods S1.

## Results

**Figure 1:**
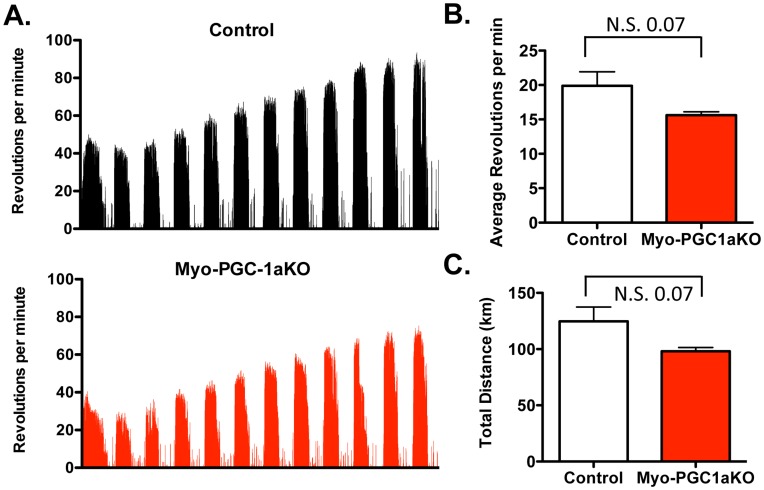
Mild decrease voluntary-wheel performance in Myo-PGC-1α animals. A.) 10 to 12 week old Myo-PGC-1αKO mice and control littermates were individually housed in voluntary running wheel cages with electronic monitoring system for 2 weeks. Tracing of wheel activity, in revolutions per minute is shown. B.) Average number of revolutions per minute C.)Total distance ran in kilometers (km). Error bars indicate *s.e.m*.; n >6 per group in all panels. * - P<0.05 compared to control.

Mice lacking PGC-1α throughout the body have numerous systemic effects, including hypermetabolism, hyperactivity, and a reluctance to exercise [35], [51], rendering them a poor model to study EIMB in skeletal muscle. Mice bearing myocyte-specific deletion of PGC-1α (Myo-PGC-1αKO mice) were thus used. The mice were generated by Cre/Lox recombination and transgenic expression of Cre with a myogenin/MEF2 promoter/enhancer construct, as previously described [35], [52], [53]. 12-week old female Myo-PGC-1αKO mice and littermate control mice were allowed to exercise in individually housed cages with hanging voluntary running wheels for 12 days. The wheel revolutions per minute were assessed over the time course using an in-cage monitoring system (Figure 1A). As shown in Figure 1, both Myo-PGC-1αKO and control mice voluntarily ran nightly, and rested daily, running approximately 10 hours/day for an approximate calculated total distance of 100 km over the 2-week period. Mice of both genotypes initially ran at 20–40 revolutions/minute, and increased their running performance over the subsequent 9 days to a plateau of approximately 60–80 revolutions/minute. Overall, the Myo-PGC-1αKO mice revealed a mild, non-statistically significant, reduction in exercise performance, as assessed by both the average revolutions per min (Figure 1B) and total distance run (Figure 1C). Therefore the Myo-PGC-1αKO mice provide a good system for assessing the effects of deleting PGC-1α in skeletal muscle without any of the negative effects of germline deletion of PGC-1α.

### Induction of OXPHOS Genes in Myo-PGC-1α Mice

**Figure 2:**
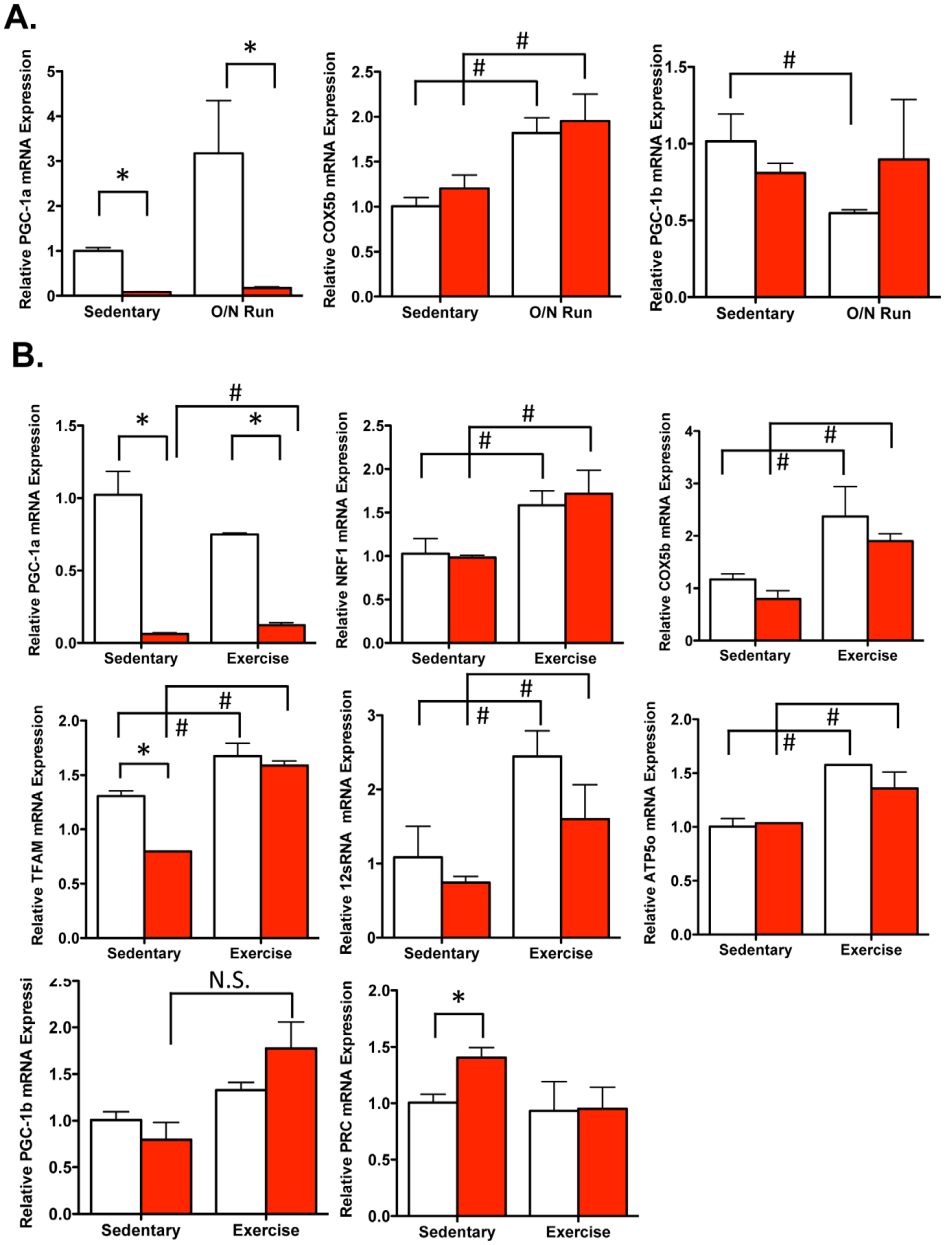
Induction of OXPHOS genes in Myo-PGC-1α animals. A.) After overnight bout of voluntary running wheel, RNA was prepared from quadriceps muscles of the Myo-PGC-1αKO (red bar) and littermate controls (white bar), and the expression of the indicated genes measured by quantitative RT-PCR. B.) After 2 week bout of voluntary running wheel, RNA was prepared from quadriceps muscles of the Myo-PGC-1αKO (red bar) and littermate controls (white bar), and the expression of the indicated genes measured by quantitative RT-PCR. Error bars indicate *s.e.m*.; n >3 per group in all panels. * - P<0.05 compared to control; # - P<0.05 compared to sedentary.

We next sought to determine whether exercise induced-genes where affected in Myo-PGC-1αKO mice after an overnight bout of voluntary wheel running. Myo-PGC-1αKO mice and control littermates were placed in individually housed cages either with wheels, or without as sedentary controls. The Myo-PGC-1αKO mice and the control mice ran overnight to a similar extent (data not shown). The next morning, quadriceps (QUADs) were harvested, RNA was isolated using the Trizol method, and the relative levels of mRNA expression of various genes were assessed by quantitative real-time PCR (qPCR). PGC-1α mRNA was induced in response to exercise ∼3-fold (Figure 2A), as has been reported [35]. Importantly, the expression of PGC-1α was almost completely absent in the Myo-PGC-1αKO animals (Figure 2A), consistent with efficient deletion of PGC-1α in the myocytes compartment, and relatively low PGC-1α expression in non-myocytic cells in skeletal muscle. Cytochrome c oxidase subunit Vb (COX5b), known to be induced by exercise, and a known target of PGC-1α, was induced in response to exercise, as expected (Figure 2A). Surprisingly, however, COX5b was similarly induced by exercise in the absence of PGC-1α (Figure 2A). No significant compensatory changes in PGC-1β expression were seen in the absence of PGC-1α (Figure 2A). We next sought to extend these findings to the response to a longer bout of exercise. Myo-PGC-1α KO and control littermates were subjected to a 12-day wheel run, again using littermate controls, and sedentary controls. Prolonged exercise induced in quadriceps of control mice the expression of COX5b, ATP synthase subunit 5o (ATP5o) as well as the nuclear respiratory factor 1 (NRF1), the mitochondria encoded 12 s RNA, and transcription factor A (Tfam) (Figure 2B), as expected. Again, surprisingly, all of these genes were induced as efficiently in the absence of muscle PGC-1α (Figure 2B). The soleus, a predominantly type I muscle, did not reveal any significant exercise-induced response (Figure S1). Taken together, these results show that PGC-1α is dispensable for the exercise-mediated induction of genes encoding key components of oxidative function and mitochondrial biogenesis.

### Normal Exercise-Induced Mitochondrial Biogenesis in Myo-PGC-1α KO mice

**Figure 3:**
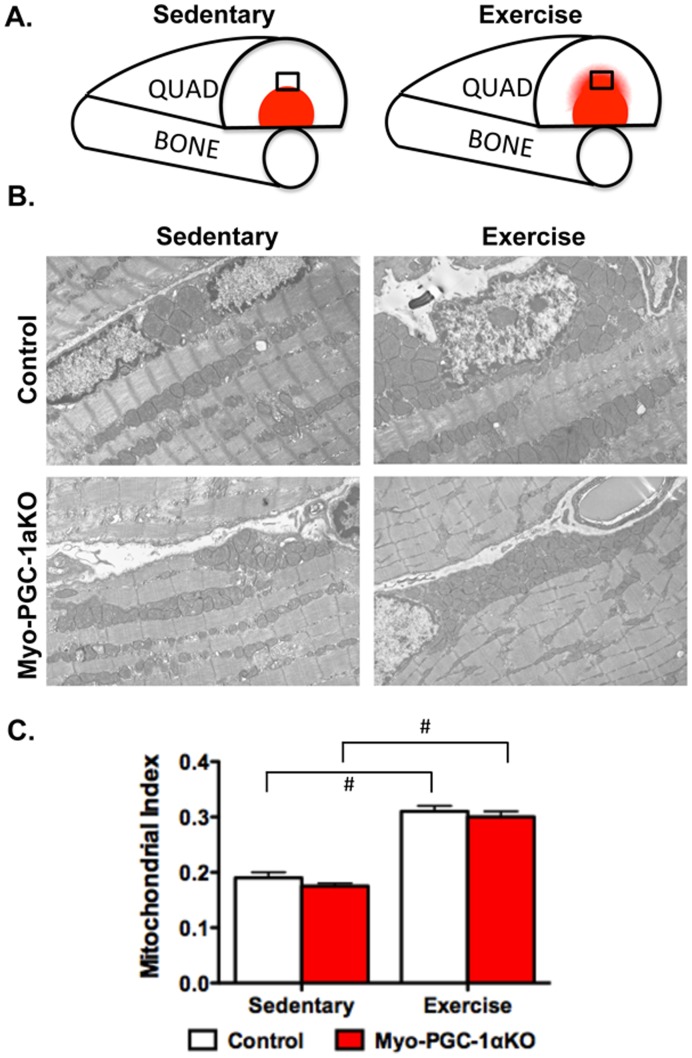
Exercise induced mitochondrial biogenesis in Myo-PGC-1α animals. A.) Schematic of mid-portion of muscle recruited in response to exercise. Shading represents oxidative portion of muscle. Black square represents region of interest (ROI) used for studies B.) Transmission electron micrographs (TEM) of transverse sections of recruited ROI of quadriceps before and after 2-week voluntary wheel run C.) Quantification of mitochondrial density from TEMsof the Myo-PGC-1αKO (red bar) and littermate controls (white bar).n>4 fields from 4 animals per group. Error bars indicate *s.e.m*.; n >3 per group in all panels. * - P<0.05 compared to control.

We next sought to evaluate mitochondrial biogenesis in response to exercise in the absence of PGC-1α. Endurance exercise recruits different muscles, and even different portions of muscles, differently [54], [55]. In the quadriceps muscle, for example, endurance exercise primarily recruits the middle portion of the muscle (Figure 3A). The deep portion in close proximity to the bone is likely used for locomotion and postural maintenance even in sedentary animals, while the superficial portion of the muscle is more likely to be recruited for low-endurance functions such as strength tasks. We thus focused our attention on the middle portion of the quadriceps (Figure 3A). Myo-PGC-1αKO and control mice were again allowed to run for 12 days on voluntary wheels, versus sedentary controls. Electron micrographs of the recruited portion of the quadriceps muscle were then imaged, and the mitochondrial density in the muscle was quantified using morphometric analyses. As shown in Figure 3B, 12 days of endurance exercise led to a marked 60% induction of mitochondrial density in this part of the muscle of wildtype run mice. This exercise-induced increase in mitochondrial density was entirely normal in the Myo-PGC-1αKO mice (Figure 3B). PGC-1α is thus dispensable for mitochondrial biogenesis in skeletal muscle in response to exercise.

### Preserved Exercise-Induced Electron Transport Complex Activity in Myo-PGC-1αKO mice

**Figure 4:**
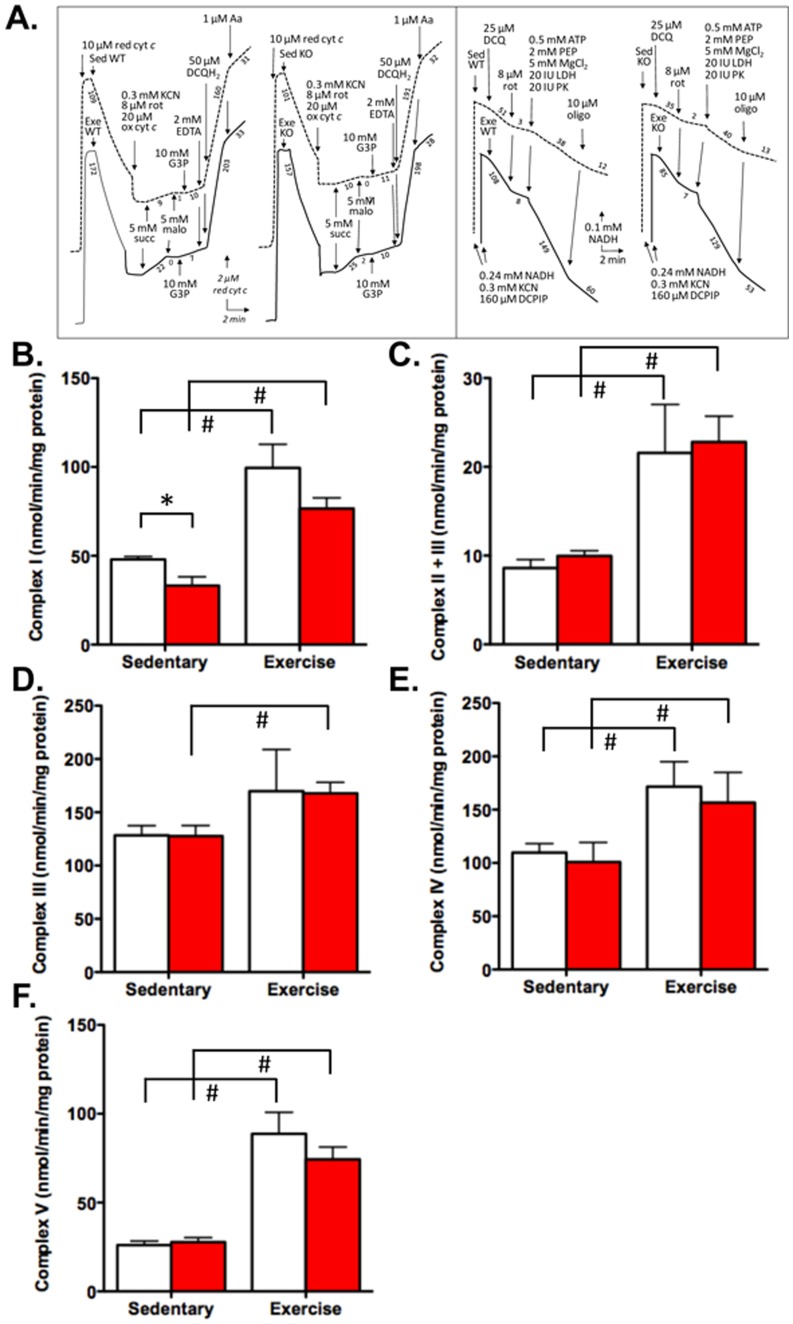
Intact Electron Complex Activity in Myo-PGC-1α in response to exercise. A.) Enzymatic traces of complex activities B.) Rotenone sensitive NADH dehydrogenase activity (Complex I). C.) Succinate-cytochrome *c*reductase activity (Complex II+III). D.) Glycerol-3-phosphate dehydrogenase + Complex III, rate dependent on glycerol-3-phosphate dehydrogenase (Complex III). E.) Cytochrome oxidase activity (Complex IV).F.) ATPase activity (Complex V). Myo-PGC-1αKO (red bar) and littermate controls (white bar). Error bars indicate *s.e.m*.; n >3 per group in all panels. * - P<0.05 compared to control; # - P<0.05 compared to sedentary.

We next sought to directly measure mitochondrial oxidative capacity in response to exercise. Myo-PGC-1αKO and control mice were again allowed to run for 12 days, after which the recruited portions of the quadriceps muscle were harvested. The activity of all 4 complexes of the electron transport chain, as well as the ATPase (Complex V) were then measured directly, using a state-of-the-art enzymatic spectrophotometric assay (Figure 4A, B, C, D, E, F) [50]. As shown in Figures 4B, C, D, E, F, exercise led to strong induction of the functional capacity of each of the 5 complexes, ranging from 30% increase (ComplexIII) (Figure 4D) to a tripling of activity (Complex V) (Figure 4F). Strikingly, this strong induction of respiratory capacity was almost entirely preserved in the absence of PGC-1α. Complex I activity after exercise was reduced approximately 25% in the Myo-PGC-1αKO (Figure 4B), but the activity was also reduced in the sedentary animals, so that the induction of activity induced by exercise was similar in both genotypes. Complex V activity was reduced approximately 15% in the exercised Myo-PGC-1αKO mice, but not in the sedentary animals (Figure 4F). The induction of the activity of the 3 other complexes by exercise was entirely preserved in the Myo-PGC-1αKO mice. PGC-1α in skeletal muscle cells is thus not required for exercise-induced adaptations of mitochondrial capacity.

## Discussion

Mitochondrial biogenesis is strongly induced in skeletal muscle by exercise [1], [2], [3]. PGC-1α potently induces mitochondrial biogenesis in skeletal muscle [11], [12], [13], and PGC-1α expression in skeletal muscle is strongly induced by exercise. Gain-of-function observations in mouse models have led to the widely accepted belief that PGC-1α mediates exercise-induced mitochondrial biogenesis [11], [17], [19]. Formal testing of this hypothesis had not been done, however. We show here, using a loss-of-function approach, that, surprisingly, PGC-1α is in fact dispensable for exercise-induced mitochondrial adaptations in skeletal muscle. This surprising finding underscores the importance of loss-of-function studies, and highlights the still incomplete understanding of the mechanisms by which exercise strongly activates mitochondrial biogenesis in skeletal muscle. The finding also cautions against concluding that elevations of PGC-1α expression concomitant with increases in mitochondrial biogenesis necessarily indicate a causal relationship. Interestingly, Uguccioni et al. showed in a muscle cell culture model that siPGC-1α failed to block the induction of many oxidative genes and function by forced contractile activity, consistent with our findings here [56].

What other mechanisms could activate exercise-induced mitochondrial biogenesis in the absence of PGC-1α? Pogozelski et al. have recently shown that p38γmitogen-activated protein kinase (MAPK) (but not p38α or β) is required for EIMB, indicating that the MAPK pathway is critical [57]. Similarly, AMP-activated protein kinase (AMPK) has been identified as important in regulating mitochondrial content in response to exercise [58]. However, we do not observe any significant changes in either total or phospho-AMPK in our Myo-PGC-1α mice (Figure S2). PGC-1α protein and message are known to be affected by both p38 and AMPK [33], [59], but our data indicate that other targets must thus also exist in this context. PGC-1β is a homolog of PGC-1α (40 and 48% identity in the conserved N-terminal activation domain and RNA binding domain, respectively) [25] that shares many, though not all, of the roles of PGC-1α. In skeletal muscle, PGC-1β can induce mitochondrial biogenesis as robustly as PGC-1α [18]. Deletion of both PGC-1α and β in skeletal muscle reduces mitochondrial function markedly more than deletion of either alone [24]. Unlike PGC-1α, however, expression of PGC-1β is not induced by exercise or adrenergic stimulation, and may in fact be decreased [40], [60]. For this reason, PGC-1β has not generally been thought to play a role in EIMB. We also did not observe compensatory increases in PGC-1β expression in the Myo-PGC-1α mice. Thus, if PGC-1β is compensating for the absence of PGC-1α, then exercise must be modulating PGC-1β post-transcriptionally, for example via adrenergic and/or p38-mediated or AMPK-mediated phosphorylation. The contribution of PGC-1β to EIMB will need to be addressed with muscle-specific deletion of PGC-1β, alone or concurrent with deletion of PGC-1α. Lastly, the hypoxia inducible factors 1 and 2 (HIFs) have also been shown to be induced in response to exercise [61]. However, HIFs likely suppress mitochondrial function and OXPHOS [62], [63]and thus are unlikely candidates to mediate EIMB.

The current study does not evaluate every form of exercise, but rather focuses on voluntary endurance exercise. This choice was guided by the extensive amount of exercise that mice perform voluntarily, as well as the strong mitochondrial biogenic response to the stimulus. The study cannot rule out, however, that mitochondrial adaptations to other forms of exercise, such as controlled endurance exercise or strength training, might be mediated by PGC-1α. Nevertheless, voluntary running in in-cage wheels robustly induces PGC-1α in skeletal muscle [35], and despite this induction, reveals no dependency on PGC-1α for the induction of mitochondrial biogenesis (Figure 3C).

It is possible, though unlikely, that PGC-1α was insufficiently deleted from skeletal muscle in our study. The myogenin/MEF2-CRE driver has been used widely, and indeed the mRNA expression of PGC-1α was dramatically reduced in all muscles tested, and especially so in the muscles used for subsequent mitochondrial experiments. Moreover, the same muscle-specific PGC-1α knockout mice have been used by us and others to highlight other phenotypes, including the existence of a muscle/beta cell crosstalk, and the requirement for PGC-1α for exercise-induced angiogenesis [35], [53]. Finally, it is highly unlikely that the few myonuclei that have retained a non-deleted PGC-1α locus could induce, at a distance, target genes throughout the myofiber. Therefore it is unlikely that any of the exercise-induced effects observed in this study are due to incomplete deletion of PGC-1α. It is also possible that ontogenic deletion of PGC-1α led to compensatory changes during development, although, as noted above, no changes were observed in the expression of the most likely candidates, PGC-1β and PGC-1-related coactivator (PRC).

Recently, a naturally occurring alternatively spliced form of PGC-1α that is truncated at exon 7 (NT-PGC-1α) has been shown to have some of the biological activity of full length PGC-1α [64], [65], [66]. The role of NT-PGC-1α in skeletal muscle is not known. Importantly, however the Myo-PGC-1α KO animals used in this study lack exons 3 thru 5 of PGC-1α, which also affects NT-PGC-1α. Therefore NT-PGC-1α is unlikely to play a role in mediating the exercise-induced responses observed on the Myo-PGC-1α KO mice.

It is interesting that, while PGC-1α is not required for EIMB (this study), or for exercise-induced changes in fiber types [67], it does appear to be critical for exercise-induced angiogenesis [35], underscoring the complexity of the muscle response to exercise, and the role of PGC-1α.Therefore, while dispensable for some aspects of exercise-induced changes, PGC-1α clearly still has a pivotal role in skeletal muscle adaptions in response to exercise.

In summary, the current study demonstrates that PGC-1α is dispensable within skeletal muscle for exercise-induced mitochondrial adaptations, in sharp contrast to the prevalent assumption in the field, and indicating that other important pathway(s) clearly exist. Exercise is a powerful intervention for the treatment of many diseases [68], [69], [70], [71], [72], [73], and exercise-induced changes on mitochondria are likely important for many of the benefits of exercise [3], [4], [5], [6]. Identifying and understanding the PGC-1α-independent pathways that mediate EIMB will thus be of great interest.

## Supporting Information

Figure S1 **Expression of OXPHOS genes in soleus of Myo-PGC-1α animals.** After 2-week bout of voluntary running wheel, RNA was prepared from soleus muscles of the Myo-PGC-1αKO (red bar) and littermate controls (white bar), and the expression of the indicated genes measured by quantitative RT-PCR. Error bars indicate *s.e.m*.; n >3 per group in all panels. * - P<0.05 compared to control; # - P<0.05 compared to sedentary.
(TIF)

Figure S2 **Phospho and Total AMPK levels in quadriceps of Myo-PGC-1α animals.** After 2-week bout of voluntary running wheel, protein was prepared from quadriceps muscles of the Myo-PGC-1αKO and littermate controls, levels of P-AMPK and total AMPK were assessed by western blotting analysis.
(TIF)

Methods S1 **Additional details on method for western blotting analysis.**
(DOCX)
