# Supplementary figures and images for: PGC-1α is Dispensable for Exercise-Induced Mitochondrial Biogenesis in Skeletal Muscle
Source: PLoS One. 2012 Jul 24;7(7):e41817. doi: 10.1371/journal.pone.0041817 (PMC3404101; doi:10.1371/journal.pone.0041817)

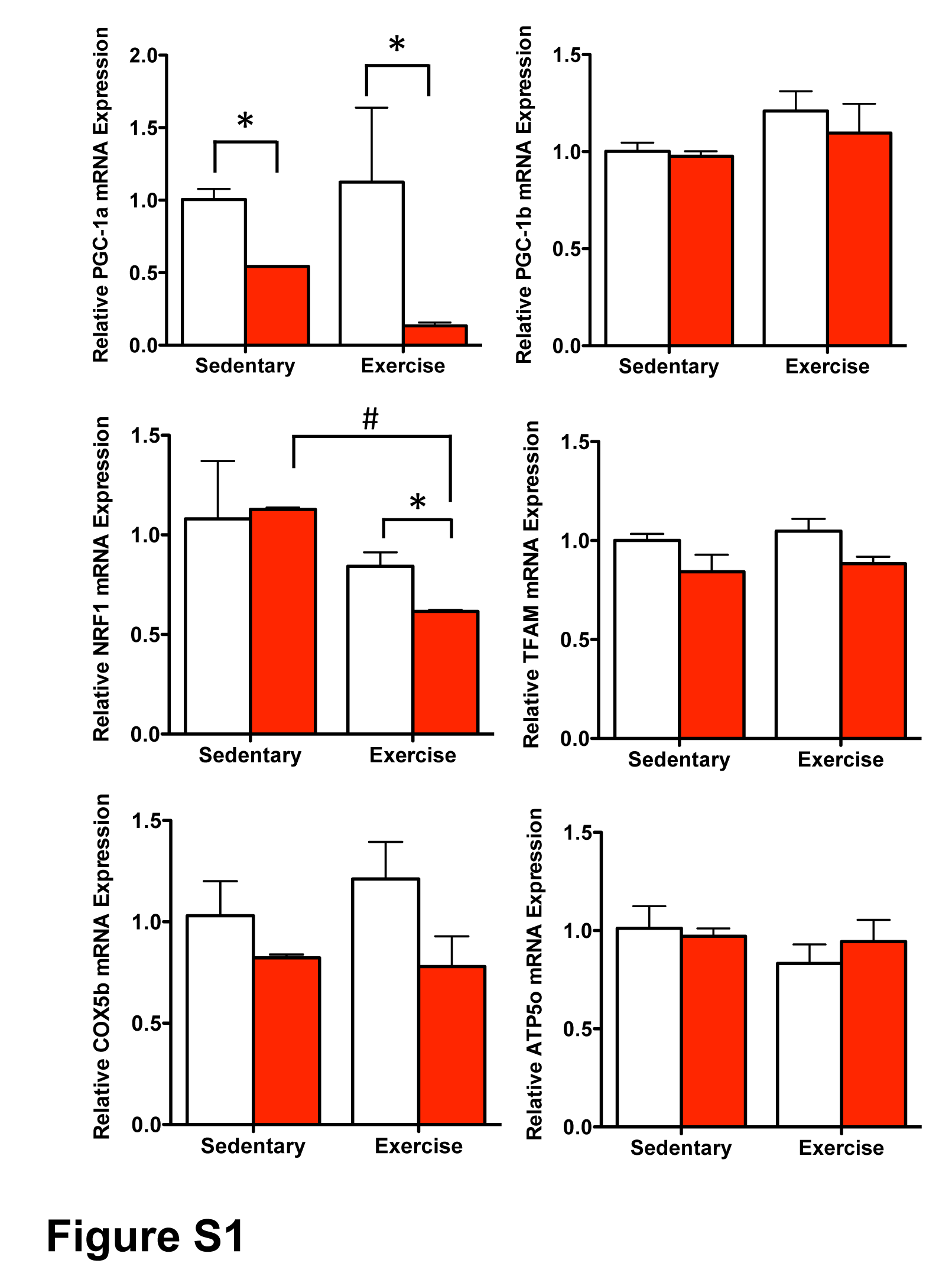

Supplement: Figure S1 — Expression of OXPHOS genes in soleus of Myo-PGC-1α animals. After 2-week bout of voluntary running wheel, RNA was prepared from soleus muscles of the Myo-PGC-1αKO (red bar) and littermate controls (white bar), and the expression of the indicated genes measured by quantitative RT-PCR. Error bars indicate s.e.m.; n >3 per group in all panels. * - P<0.05 compared to control; # - P<0.05 compared to sedentary. (TIF) [file pone.0041817.s001.tif]

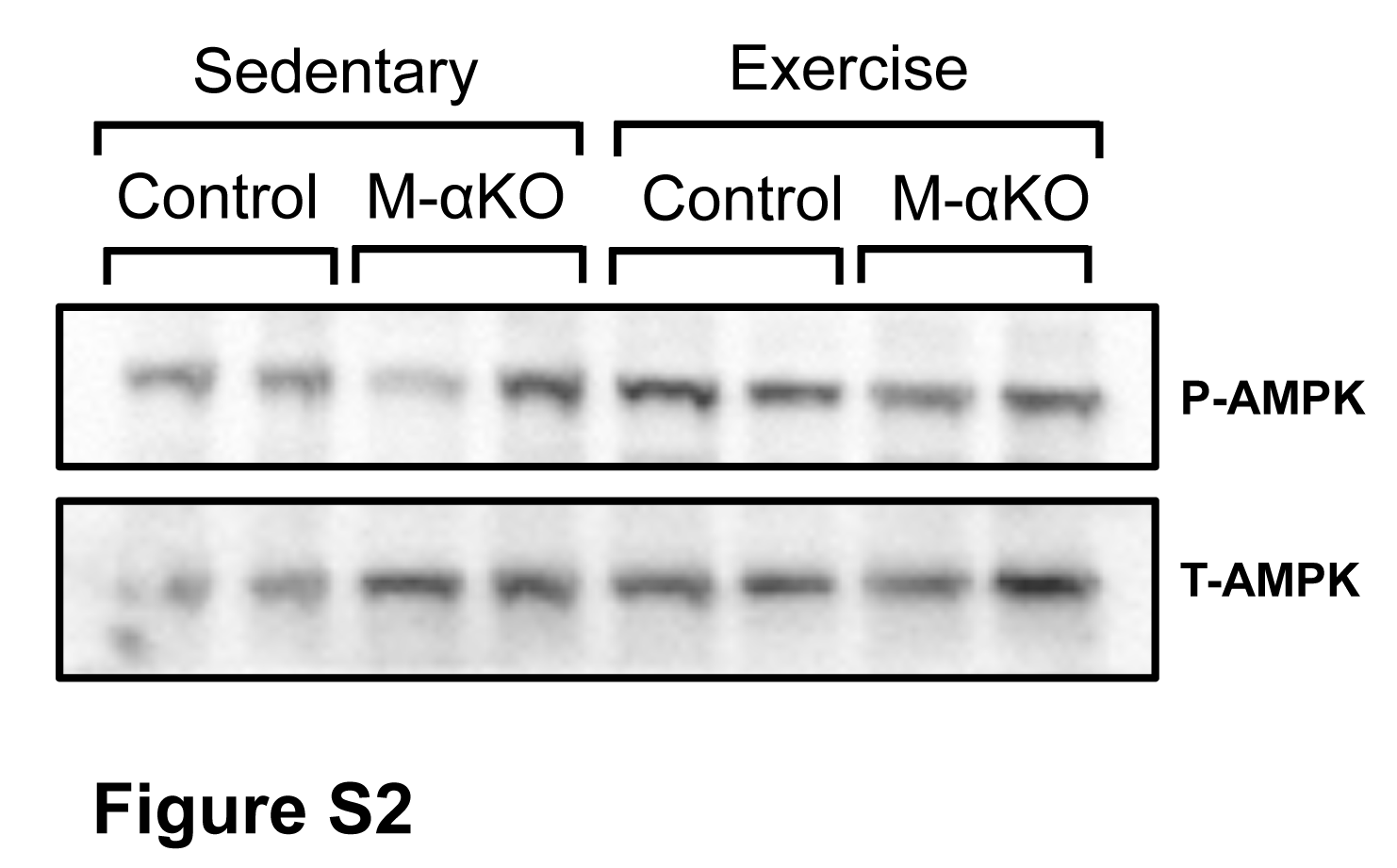

Supplement: Figure S2 — Phospho and Total AMPK levels in quadriceps of Myo-PGC-1α animals. After 2-week bout of voluntary running wheel, protein was prepared from quadriceps muscles of the Myo-PGC-1αKO and littermate controls, levels of P-AMPK and total AMPK were assessed by western blotting analysis. (TIF) [file pone.0041817.s002.tif]
