# Supplementary material for: PGC-1α is Dispensable for Exercise-Induced Mitochondrial Biogenesis in Skeletal Muscle
Source: PLoS One. 2012 Jul 24;7(7):e41817. doi: 10.1371/journal.pone.0041817 (PMC3404101; doi:10.1371/journal.pone.0041817)
Supplement: Methods S1 — Additional details on method for western blotting analysis. (DOCX) [file pone.0041817.s003.docx]

**Supporting Methods Information**

Western Blotting – Total protein was isolated from mouse tissue using modified RIPA supplemented with protease and phosphatase inhibitors. Protein extracts (~30 µg) were subjected to electrophoresis on 4-12% Bis-Tris gels (Invitrogen) and transferred to nitrocellulose membranes (BioRad) for Western blot analysis. Total AMPK α-pan and Phospho AMPK-α (Thr172) antibodies were purchased from Millipore and Cell Signaling, respectively. Bands were detected by ECL procedure following the manufacturer’s instructions.
